# Delineating risk zones and evaluation of shelter centres for flood disaster management along the Pahang River Basin, Malaysia

**DOI:** 10.4102/jamba.v10i1.501

**Published:** 2018-04-23

**Authors:** Anizan Isahak, Mohammad I.H. Reza, Chamhuri Siwar, Shaharuddin M. Ismail, Norela Sulaiman, Zulkifli Hanafi, Mohd S. Zainuddin, Mohd R. Taha

**Affiliations:** 1 School of Environmental and Natural Resources Science, Universiti Kebangsaan Malaysia, Malaysia; 2 Institute for Environment and Development, Universiti Kebangsaan Malaysia, Malaysia; 3 Faculty of Architecture and Built Environment, Infrastructure University Kuala Lumpur, Malaysia

## Abstract

Shelter centres are important locations to safeguard people from helpless situations and are an integral part of disaster risk reduction (DRR), particularly for flood DRR. The establishment of shelter centres, and their design based on scientific assessment, is crucial. Yet, they are very much related to the geographic location, socio-economic conditions and the livelihoods of the affected communities. However, many parts of the developing world are still lagging behind in ensuring such scientific design. Considering the flood disaster in 2014 that affected the residents living along the Pahang River Basin, in this study we delineate the communities at risk and evaluate the existing shelter centres to determine how they reduce people’s vulnerability to the risks associated with rural and urban landscapes. We used spatial analysis tools to delineate risk zones and to evaluate existing evacuation systems. A flood disaster risk map was produced to determine which communities are living with risks. Subsequently, the distribution of shelter centres examined whether they are able to support people living at the flood risk zones. These centres were also evaluated using a set of international guidelines for effective disaster shelters. This reveals that the number of shelter centres is not adequate. The designation and designing of shelter centres are not being done scientifically. The maps produced here have a lot of potential to support disaster management decisions, in particular site selection and the prioritisation of centres. The study concludes with a set of guidelines and recommendations for structural and non-structural measures, such as alternative livelihoods and the potential of ecotourism, which may improve the resilience among flood-affected communities; and the decision-making process for the overall flood DRR initiatives.

## Introduction

Shelter centres are most important places to safeguard people from the helpless situation and they are an integral part of disaster risk reduction (DRR), particularly for the flood DRR. It is one of the key sectors in post-disaster scenarios which aims to provide disaster-affected individuals and communities with assistance in the form of temporary protection that may safeguard them from severe loss that may have been caused because of disasters. In fact, human designs that are incompatible with the prevailing natural perturbation may create disastrous consequences (Bashawri, Garrity & Moodley 2014).

In Malaysia, the recent flood event (which occurred between December 2014 and January 2015) has affected many people by destroying their houses, properties and livelihoods, particularly in the northeastern part of Peninsular Malaysia where human activities have modified the natural landscapes into cultural landscapes. These anthropogenic activities are among the proximate causes that make the area prone to recurrent flood events. As a consequence, the demand for shelter centres to safeguard people’s lives during disasters is getting higher. It is also evident that many of the shelter centres were also flooded during the recent flood. Therefore, it is important to evaluate the efficiency and design that is suitable for use as shelter centres. From a practical point of view, designing and building disaster-resilient communities are much more effective in reducing the impact of a disaster than merely providing post-disaster support (Felix, Branco & Feio 2013). However, the culture of rural and urban livelihood differs greatly, and resilient approaches should be fashioned and designed to accommodate these cultural variances.

Structural measures are taken to be any physical structure constructed to reduce or avoid possible impacts of hazards, or the application of engineering techniques to achieve hazard-resistance and resilience in structures or systems. Dams, flood levies, ocean wave barriers, flood- and earthquake-resistant construction, and evacuation shelters are some of the common structural measures for DRR. Non-structural measures are measures related to the physical management in reducing flood risks and include land planning, building codes, design standards, construction practices, pluvial control, sediment management, elevation, deforestation control, maintenance, the conservation of wetlands and natural storage. When structural and non-structural measures are implemented in an effective and sustainable manner, the community will have achieved a high level of structural resilience to flood hazards.

Community resilience, according to Norris et al. (2008), is the ability of communities to withstand hazards. Central to the idea of resilience is the capacity to anticipate risk, limit impact and recover rapidly through adaptability. In the aftermath of adversity, a resilient community quickly returns to its functionality, both culturally and economically, utilising its resources and social connectedness. To assess the level of resilience in a community, it is therefore important to determine the assets and vulnerabilities of community livelihood? One of the ways for a community to cope with disasters is through designing, building and maintaining structures that improve with learnt lessons and shared practices. This practice will strengthen the community to be better prepared for combating potential disaster threats. The Sendai Framework for DRR (UNISDR 2015) spells out the following four areas of action:

- Priority 1: Understanding disaster risk.
- Priority 2: Strengthening disaster risk governance to manage disaster risk.
- Priority 3: Investing in DRR for resilience.
- Priority 4: Enhancing disaster preparedness for effective response, and to ‘Build Back Better’ in recovery, rehabilitation and reconstruction.

This study investigates the structural and non-structural measures for flood DRR and identifies effective designs that may help the community to be resilient to flood disasters, with a particular focus on priorities 3 and 4 of the above Sendai Framework for disaster risk reduction (SFDRR) priority areas. The study area is located at the communities flanking the riverbanks of the central part of the Pahang River. A sustainable livelihood approach was taken to identify the types of structural and non-structural measures to be taken for disaster risk management. Ecotourism that is compatible with the environmental conservation of the river floodplain and the use of sustainable building material such as bamboo featured as good options for disaster risk management strategies. This article reports on the livelihood assets that support ecotourism in the area, as well as suggestions for structural designs and bamboo value creation, measures that are appropriate to the development of ecotourism in the area (Designboom 2013; Kalema 2013).

At the same time, identifying disaster risk structures and designing resilient structures in urban cultural landscapes is important for a long-term flood disaster mitigation plan for the river basin. It can be assumed that designing disaster-resilient structures and eco-friendly landscapes will provide more strength for effective flood disaster risk management in river basins having anthropogenic influences, like the Pahang River Basin. This study intends to build up useful and resilient structures’ to reduce the risk of disasters. The specific objectives of this research are twofold: to identify the flood risk zones and to evaluate the existing shelter centres in relation to the infrastructure conditions in urban and rural settings in the Pahang River Basin. This is to assess suitable and effective types of flood disaster resilience structural designs for urban areas in the study area. It is also assumed that the results of the study may be able to support the decision process of the policy implementation for flood DRR.

## Study area

### Pahang River Basin

The Pahang River, with a length of 459 km, is the longest river running through the state of Pahang, of Peninsular Malaysia. Originating from the Mountain Titiwangsa, Jelai and Tembeling rivers, they meet at Kuala Tembeling, at a confluence 300 km away from the estuary of the Pahang River (Kuala Pahang). The Pahang River drains into the South China Sea. The Pahang River Basin comprises almost all the districts of the state of Pahang, containing the Cameron Highlands, Temerloh, Pekan, Lipis, Jerantut, Bera, Maran and a part of Kuantan. The heavy rainfall, as a result of the northeast monsoon, occurs in this area from November to March of every year, and the lower areas of the basin experience frequently overflows beyond the danger level (Pan et al. 2011), which is an issue of great concern due to its cascading effects.

### Kuantan River Basin

The Kuantan River runs from Sungai Lembing through Kuantan City before it flows out to the South China Sea. The elevation range is from 0 m at the mouth of the watershed to 1511 m above sea level at the highest tip of the northwestern watershed. The river basin contains several main tributaries, which drain the rural, agricultural, urban and industrial areas of Kuantan.

**FIGURE 1:**
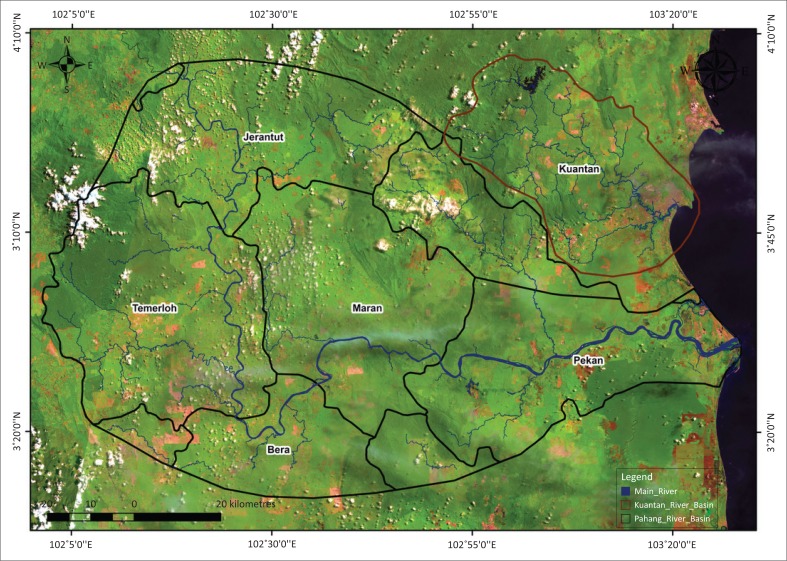
Pahang and Kuantan river basins of Peninsular Malaysia, showing the study area.

The shelter centres of Temerloh, Kuantan and Pekan were visited, and their location coordinates noted with a handheld GPS. An extensive house-to-house visit of the severely affected community was undertaken, and randomly selected residents from each area were interviewed (see Figure 1). Comprising a number of sub-districts and villages in each district, the study mainly focused on the worst affected rural and urban areas during the floods.

## Methodology

### Data accumulation

**Flooding data of 2014:** The extensive data of flooding regime of the 2014 flood were collected through extensive field work in the study areas. The height and spatial extent of flood inundation were noted and noted using a GPS. Later, based on this information, three scales – high, moderate and low flood inundation regimes – were classified.

**Shelter centre data:** A list of shelter centres in the study area were collected from the district offices of Pekan, Kuantan and Temerloh. These shelter centres were visited, and coordination points were recorded using a handheld GPS. Later these points were placed on the digitised risk map.

**House location data:** The house location data (shapefile format) were elicited from a land-use map obtained from the Department of Survey and Mapping, Malaysia (JUPM). Later these house location points were plotted on the risk map.

### Digitising data layers

Digitisation in GIS is a process of ‘tracking’, in a geographically correct way, information from images or maps. The gross flood disaster risk paper map was collected from the Department of Irrigation and Drainage (DID) in Malaysia. This map was used as the base map for the digitising process (scale 1:200 000) and the part of the thematic paper map was scanned using a high-performance (HP) scanner. The scanned maps were saved in the Tagged Image File Format (TIFF) form and ArcGIS 9.3 was used for the digitising process. Before the digitising process, the data layers were georeferenced, which relies on the coordination of points on the scanned image (data to be georeferenced) with points on a geographically referenced data map. By ‘linking’ points on the image with those same locations in the geographically referenced data, a polynomial transformation was created that converts the location of the entire image to the correct geographical location.

After georeferencing each image, a digitisation process was performed using ArcGIS 9.3. Digitising is the process of converting a paper map or image data to vector digital data. In ArcGIS, the point, line or polygon of an image can be redrawn following the source data. In this case, redrawing was done through controlling a cursor and using a computer mouse; sample vertices were drawn to define those attributes. The structures of these vertices can be seen in the images of the data layer in its editable form where those features are assigned additional spatial and non-spatial attributes. Through this process, digital versions of maps were generated, which have an attribute table associated with them. The digitising process was started by creating a new (vector) layer in ArcCatalog, and then adding features to them in ArcMap (ArcGIS 9.3 Desktop). Editor Toolbar was used for the digitisation of the data layer in the newly created vector layer or shape file.

### Mapping procedure

At first, all data layers were geocoded and georeferenced in the same coordination system. These data layers were integrated, and they were then overlaid in a GIS platform. ArcGIS 9.3 was used to integrate data layers for subsequent analyses. All the data layers were overlaid using the Spatial Union in the GIS platform.

### Sphere Project analysis

Overall infrastructural design and specifically designated shelters were assessed. The present study focused on several evacuation centres in close proximity to the town of Temerloh, Malaysia, which was assessed in terms of the location of evacuation centres, site planning, the use of appropriate materials, facilities, spatial layout and capacity. The study concludes with a section for further improvements and suggestions for policymakers.

The Sphere Project’s Minimum Standards in Humanitarian Response was used as a benchmark. These standards are useful in a range of response scenarios for displaced and non-displaced populations, including temporary or transitional household shelters, temporary accommodation with host families or temporary communal settlements comprising planned or self-settled camps, collective centres, et cetera (The Sphere Project 2011). A social survey was conducted to evaluate the livelihood assets and vulnerabilities as well as the acceptance of bamboo as a sustainable construction material as a flood risk reduction strategy. Ecotourism livelihood assets were evaluated as the basis for further development of the ecotourism concept for DRR through livelihood enhancement strategies.

### Ecotourism potential analysis

The ecotourism potential of the study area has been evaluated and considered social, cultural, environmental and economic importance and potentials of the locally available material (i.e. bamboo). Similar evaluations have been applied through the work of many researchers (e.g. Akinlabi & Adeniran 2014; Bansil et al. 2015; Jiang 2008). Here, we have selected the indicators and evaluated their potential through direct field observations and interviewing local community leaders and members.

## Results and discussion

### Flood risk zones and structures

**FIGURE 2:**
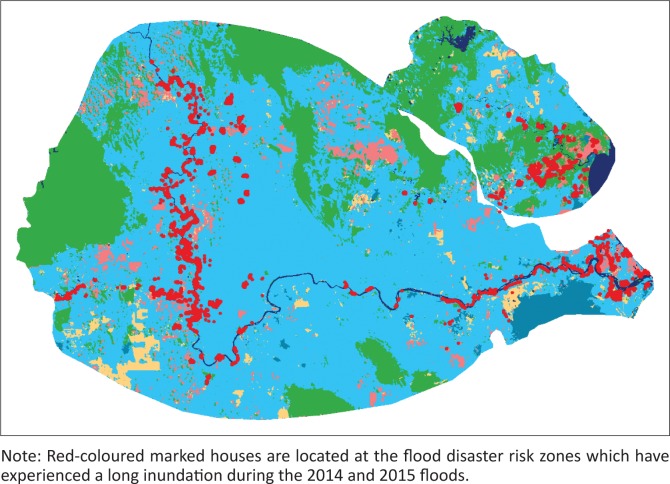
Distribution of the population that are residing in the flood disaster risk zones along the Pahang and Kuantan river basins.

Using satellite images and GIS technology, an approximation of a flood risk map was produced (Figure 2). The 2014 flood (which occurred between December 2014 and January 2015) affected populations. Their houses have been visualised in this map and showed that a considerable proportion of the community has been residing in the flood disaster risk zones in the Pahang and Kuantan river basin areas.

**FIGURE 3:**
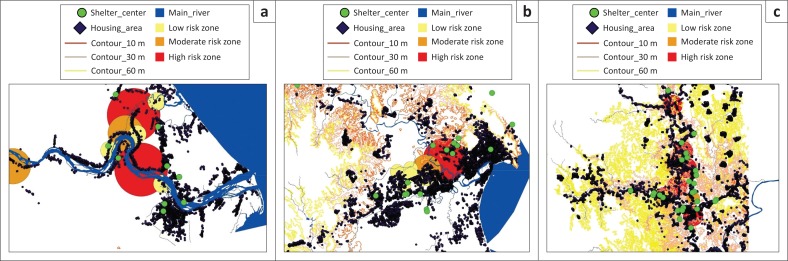
The flood disaster-affected population and shelter centres in (a) Pekan, (b) Kuantan and (c) Temerloh.

Additionally, Figure 3 shows the households that are located in the flood risk zones. These maps show the flood risk zones according to the degree of risk and the location of houses in these areas. Simultaneously, the shelter centres (in green circles) are visualised. The analyses revealed that the proportion of the shelter centres versus the affected population is not adequate. On the other hand, it is clear that the designing of the shelter centres is not being scientifically done. In general, a similiar case is found in Temerloh, Pekan and Kuantan. More specific analyses can be made using this mapping. This map also clearly shows the areas that need more shelter centres to support the disaster-affected communities. The government should take action on a priority basis to designate and design more shelter centres in the high-risk zones to save communities from the potential threats in the coming monsoonal period.

### Evaluation of shelter centres

Evacuation centres are the main lifesaving shelters during flood events. A socio-economic understanding can allow centres to be specifically equipped with the necessary facilities and functionalities. Having assessed the evacuation centres in the vicinity of Temerloh, based on the Sphere Project guidelines, the study concluded that not much development is needed in terms of structural strength but rather the design, management strategies and logistics that are involved in using evacuation centres. Recommendations for further improvement in the flood resilience of evacuation centres in the area include input from and action by all parties, including the local community, city council, academics and the private sector. Up-to-date data and information can be made available to comprehensively understand the geographical and socio-economic changes, in terms of land use, landform and local people’s needs. An integrated approach which utilises GIS technology should be used to understand the affected sites and the best locations for evacuation sites. While the use of LiDAR technology for mapping structures and landscapes may be expensive at this point in time, the high geospatial accuracy exhibited by such maps should be considered as an investment to give accurate guidance on land-use planning for DRR (Serafica 2013). Schools are prioritised as evacuation centres for several good reasons. However, the themes and objectives of the Sendai Framework for DRR must be put into action, especially the concept of ‘Build Back Better’. Schools and other buildings that have been identified as suitable evacuation centres should be designed to be adaptable and multifunctional. The Sphere Project emphasises safe public design and construction. Temporary and permanent public buildings such as schools should be constructed or repaired to be disaster-resilient. Accessibility for those with mobility, visual or communication disabilities must also be considered. It should also be considered whether it is necessary to build permanent evacuation centres instead of relying on public buildings. If not, more public buildings and public spaces should be designed to serve as evacuation centres to eliminate the sole dependency on schools.

For post-disaster reconstruction, many shelter home designs fail to be practical because the designs were not made in consultation with the community and with their livelihoods in mind (Goodier 2015). Priority 4 of the Sendai Framework stipulates that flood risk management has to take into account how post-flood structural design can be used and improved effectively by involving communities. Resilient structures are designed in such a way as to reduce costs and the time required to rebuild and reuse should it be flooded. Alternatively, finishes can be designed to be removable or sacrificial and easily replaced in the zone affected by flooding. The design should be culturally appropriate, and preferably involve the participation of local community members at the construction stage. Robust materials and finishes should be used.

### Resilient structure for rural landscapes

**FIGURE 4:**
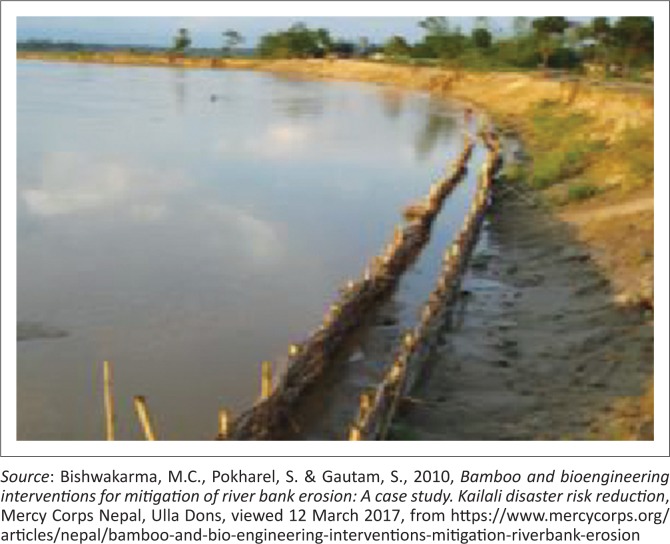
Bamboo crib wall constructed to prevent bank erosion in Nepal.

It is different while the sustainable and resilient structural designing perform for the rural landscapes. Generally, robust structural materials should have a coping capacity with the surrounding environment. The materials used should take costs, availability and ease of transport into consideration. In a life cycle assessment study that compares local and global materials for constructing shelters, Escamilla and Harbert (2015) found that although global materials such as steel and concrete are generally more superior in terms of providing efficient structures that can resist natural hazards, these materials incur a much higher embedded energy than local materials such as bamboo and wood. Local materials have the added advantage of having a higher potential for low environmental impacts and costs (Song, Mithraratne & Zhang 2016). The design consideration is of the utmost importance in using local materials so as to provide structures that resist natural hazards. Among all the materials studied, bamboo-based shelters incur the lowest impact and cost pre-functional unit. Locally in Malaysia, wood has become an unsustainable and costly material as there are now many restrictions for logging, as well as environmental issues. Bamboo has traditionally been used for the construction of temporary shelters. In recent years, rural communities in many countries emphasise the use of sustainable construction materials, such as bamboo to mitigate floods (see Figure 4; Bishwakarma, Pokharel & Gautam 2010). However, the local Malaysian populace has, in effect, lost the skills of bamboo construction because of the overdependence on global materials as a result of the nation’s development. Although bamboo can be found all over the country, the accessibility for the utilisation of bamboo as a construction material is limited. It is apparent that with proper planning for skills building in bamboo construction, and a concerted effort to plant bamboo species for construction, bamboo can be the sustainable material of choice for disaster reconstruction for the country in the foreseeable future.

### Ecotourism for livelihood enhancement

Structural measures for DRR are often only effective when the design and construction of the structures are conducted in consultation with and the involvement of the community, who are well informed of flood disaster risks and whose livelihoods may be enhanced (or not threatened) by these measures. These measures therefore need to be culturally acceptable to the community in the design and location. Floodplains, with attendant natural beauty and natural resources, can be utilised for ecotourism, thereby enhancing the community’s income and economy. The social and livelihood capitals of the village of Kg. Mengkarak, which is situated within the high flood-risk zone along the Pahang River Basin, has been studied. Table 1 lists the livelihood assets related to ecotourism potential for this small village on the banks of the Pahang River.

**TABLE 1:** Livelihood assets that can contribute to the development of ecotourism in Kg. Mengkarak, Pahang.

Livelihood assets	Sub-category	Description
Social	Social network or social capitals	A community association called Kelab Warisan Sg. Pahang was formed in 2015 to run the ecotourism venture Support from the local village leader and his committee (JKKK) An established government programme on homestays, named Kampungstay programme An information centre administered by community members to be built
	Historical assets	Pahang River is known to be the first landing site for Achehnese Muslims Sultans used Pahang River as connecting routes to southern states Several archaeological sites
	Cultural assets	Traditional spinning top industry and activity
Natural	Geological assets	Sedimentary rocks along riverbanks
	Geomorphological assets	Riverbank landscapes Wetlands
	Flora and fauna assets	Forest plant species Bamboo species Reared patin fish and others
Bamboo value creation	Floating structures	Bamboo floating houses Bamboo floating bridges Bamboo catamarans
	Soil erosion guards	Natural bamboo plants along riverbanks Bamboo crib walls
	Bamboo houses and other products	Bamboo shelters that can be turned into homestays during normal times Bamboo gateways Bamboo furniture, crafts and other products

It is evident from Table 1 that the area has a lot of social and livelihood capitals that have a high potential to support social entrepreneurship (Akinlabi & Adeniran 2014; Bansil et al. 2015). The community of this area may take effective initiatives for ecotourism and other sustainable initiatives to enhance their livelihoods. These initiatives will certainly enhance the community resilience to combat future potential threats from floods.

## Conclusion

It is evident that many places in the Malayan peninsula have become highly vulnerable to the flood hazards which have become regular occurrences. In particular, this study provides specific study-related results on various aspects of flooding as well as an evaluation of the flood shelter centres along the Pahang River Basin. In fact, the study is applicable to many such disaster-prone areas in the region.

The delineation method of flood risk zones is particularly important for the DRR decision-making process as well as for risk reduction initiatives. Risk is closely related to the hazards and vulnerability of the people. Here, flood areas, housing and existing shelter centres are integrated. Therefore, the risk zones are delineated with a maximum accuracy through this model. It is also clear which areas demand immediate initiatives for preventive and mitigation measures including the establishment of shelter centres before the next flood event occurs. However, it is also important to develop an integrated plan that takes the entire river basin into account.

Some aspects of structural measures and resilience related to DRR have been studied. It is evident that the design and planning of shelter centres were not based on scientific assessments. The methodology showed in this study may be used as a decision-supporting tool for identifying areas that need shelter centres. The method used to evaluate shelter centres can be used to evaluate the potential locations or infrastructures for identifying and designating new shelter centres. However, it is also important to proceed with the tasks on a priority basis.

This study concludes that structural resilience depends on the availability of information, public participation, community resilience and the use of soft engineering solutions. Locally available, cost-effective and sustainable construction materials and structures that mimic natural systems should be used. Given the appropriate and adequate social support, communities can build up and manage resources to better deal with future hazards. The involvement of communities in these initiatives may enhance community resilience and uplift their capacity for combating disasters and reducing their vulnerabilities. Ecotourism can be an alternative initiative that will enhance their resilience as well as social capital towards DRR.
